# Mechanism of Reduced Susceptibility to Fosfomycin in* Escherichia coli* Clinical Isolates

**DOI:** 10.1155/2017/5470241

**Published:** 2017-01-19

**Authors:** Yasuo Ohkoshi, Toyotaka Sato, Yuuki Suzuki, Soh Yamamoto, Tsukasa Shiraishi, Noriko Ogasawara, Shin-ichi Yokota

**Affiliations:** ^1^Department of Microbiology, Sapporo Medical University School of Medicine, Chuo-ku, Sapporo 060-8556, Japan; ^2^Department of Clinical Laboratory, NTT East Sapporo Hospital, Chuo-ku, Sapporo 060-0061, Japan

## Abstract

In recent years, multidrug resistance of* Escherichia coli* has become a serious problem. However, resistance to fosfomycin (FOM) has been low. We screened* E. coli* clinical isolates with reduced susceptibility to FOM and characterized molecular mechanisms of resistance and reduced susceptibility of these strains. Ten strains showing reduced FOM susceptibility (MIC ≥ 8 *μ*g/mL) in 211 clinical isolates were found and examined. Acquisition of genes encoding FOM-modifying enzyme genes (*fos* genes) and mutations in* murA* that underlie high resistance to FOM were not observed. We examined ability of FOM incorporation via glucose-6-phosphate (G6P) transporter and* sn*-glycerol-3-phosphate transporter. In ten strains, nine showed lack of growth on M9 minimum salt agar supplemented with G6P. Eight of the ten strains showed fluctuated induction by G6P of* uhpT* that encodes G6P transporter expression. Nucleotide sequences of the* uhpT*,* uhpA, glpT*,* ptsI*, and* cyaA* shared several deletions and amino acid mutations in the nine strains with lack of growth on G6P-supplemented M9 agar. In conclusion, reduction of* uhpT *function is largely responsible for the reduced sensitivity to FOM in clinical isolates that have not acquired FOM-modifying genes or mutations in* murA*. However, there are a few strains whose mechanisms of reduced susceptibility to FOM are still unclear.

## 1. Introduction

*Escherichia coli* is a causative agent of uncomplicated urinary tract infections in immunocompetent hosts and opportunistic infections in immunocompromised hosts. In recent years, fluoroquinolone-resistant and/or extended spectrum *β*-lactamase- (ESBL-) producing* E. coli* strains have been frequently isolated from such patients [1–4]. In addition, these* E. coli* resistant strains occasionally show cross-resistance to aminoglycosides [5]. Thus, these multidrug-resistant* E. coli* have an impact on the selection of therapeutically effective drugs.

Because of this serious concern, the use of fosfomycin (FOM), an antibiotic as a bacterial cell wall synthesis inhibitor developed 40 years ago, has been reevaluated against drug-resistant bacteria, especially* E. coli *[6, 7]. Since FOM has a unique mode of action that differed from other antibiotics, it is expected to display little cross-resistance to other antimicrobial agents.* E. coli* is the most frequent causative pathogenic bacterium of acute cystitis [8, 9]. For instance, trimethoprim/sulfamethoxazole and FOM are recommended as the first-line treatment in acute uncomplicated urinary tract infections according to the US guideline [10]. On the other hand, the guideline of the Japanese Association for Infectious Diseases and Japan Society for Chemotherapy recommends fluoroquinolones as the first-line drug and FOM as the second-line drug [11].

FOM inhibits UDP-*N*-acetylglucosamine enolpyruvyl transferase (MurA), an enzyme involved in the synthesis of the essential peptidoglycan component,* N*-acetylmuramic acid [12]. FOM is incorporated into the bacterial cells via glucose-6-phosphate (G6P) transporter UhpT and* sn*-glycerol-3-phosphate (G3P) transporter GlpT [13, 14]. UhpT is upregulated by exogenously added G6P [15–17]. It is thus recommended that the addition of G6P to a growth medium is used in the measurement of FOM MIC.

High resistance to FOM primarily occurs by the acquisition of glutathione* S*-transferase genes, such as* fosA*,* fosA2 to fosA5*,* fosC*,* fosC2*,* fosB*,* fosB2*,* fosX*, and* fosKP96* found in various bacteria [19, 21–26], and mutation(s) in* murA* gene [18, 24, 27, 28]. Furthermore, mutations in the transporter genes,* uhpT* and* glpT*, and genes encoding proteins regulating* uhpT* expression, such as* uhpA*, reduce the susceptibility to FOM [18, 19, 27–29]. In addition, expression levels of UhpT and GlpT are positively regulated by cyclic AMP (cAMP) [16, 17]. The levels of cAMP are controlled by phosphoenolpyruvate-protein phosphotransferase I, encoded by* ptsI*, and adenyl cyclase, encoded by* cya A*, and mutations in these genes result in reduced susceptibility to FOM [29–31].

Nevertheless, several surveillance studies report that the rate of emergence of* E. coli* isolates showing FOM resistance or reduced FOM susceptibility has been markedly low [7, 9, 32]. Although spontaneously mutational rate to acquire FOM resistance is high, FOM resistance confers biological costs, such as reduced cell growth rate in Gram-negative bacteria [29, 33]. This indicates that FOM continues to be an effective agent against* E. coli *infections. However, the overall up-to-date status of FOM resistance needs to be continuously surveyed and its molecular characteristics have to be understood to prevent future emergence and increase of multidrug-resistant* E. coli *with FOM resistance in the clinic. In this study, we screened* E. coli* clinical isolates from Japan showing resistance or reduced susceptibility to FOM and identified the molecular mechanisms of their reduced susceptibility.

## 2. Materials and Methods

### 2.1. Bacterial Strains

*E. coli* clinical isolates (211 strains) were collected in the years 2008-2009 as described previously [4, 5]. These were identified and stocked in Sapporo Clinical Laboratory, Inc. (Sapporo, Japan). These strains were collected from a variety of clinical specimens in almost entire area of Hokkaido Prefecture, Japan. This study was approved by the review boards of the relevant institutions. The strains were isolated from the following clinical specimens: urine (*n* = 87, 41.0%), catheter urine (*n* = 76, 35.8%), sputum (*n* = 15, 7.1%), stool (*n* = 7, 3.3%), vaginal secretion (*n* = 6, 2.8%), pus (*n* = 3, 1.4%), aspiration tube (*n* = 3, 1.4%), drainage tube, intravenous hyperalimentation catheter tube, rhinorrhea (two strains from each type of specimen, 0.9%), ascites, anal gland fluid, decubitus, injury site, intestinal juice, stoma, PEG insertion site, pharynx fluid, and synovial fluid (one strain from each type of specimen, 0.5%). Identification was performed using the MicroScan WalkAway 96 system (Beckman Coulter, Tokyo, Japan).* E. coli* strain ATCC25922 was obtained from American Type Culture Collection (Manassas, VA).

### 2.2. Antibiotic Susceptibility

FOM, imipenem (IPM), and ceftazidime (CAZ) were provided by Meiji Seika Pharma (Tokyo, Japan), MSD (Tokyo, Japan), and Glaxo SmithKline (Tokyo, Japan), respectively. Minimum inhibitory concentration (MIC) was determined by broth microdilution method or agar plate dilution method according to the recommendations of the Clinical and Laboratory Standards Institute (CLSI) with breakpoints according to CLSI guidelines [34]. The MIC of FOM was measured by the agar dilution method in the absence of G6P, in the presence of 25 *μ*g/mL G6P, and in the presence of 25 *μ*g/mL G6P and 2 mM cAMP.

### 2.3. Measurements of Carbohydrate Phosphate Transporter Activity

*E. coli* cells were cultured for 24 h in Muller–Hinton broth and harvested by centrifugation. The cell pellet was washed twice with saline.* E. coli* cell suspension with saline was then used to inoculate to M9 minimum salt (Becton Dickinson, Franklin Lakes, NJ) agar supplemented with 0.2% G6P or 0.2% G3P [18]. Cell growth was observed after incubation for 24 h in the case of G6P and for 48 h in the case of G3P.

### 2.4. Genetic Analysis

DNA was isolated using DNeasy Kit (Qiagen, Hilden, Germany) according to the manufacturer's instructions. Polymerase chain reaction (PCR) was performed using KAPATaq Extra HotStart Ready Mix with dye (NIPPON Genetics, Tokyo, Japan). Serogroups [35, 36] and phylogenetic groups [37] were determined by PCR. Multilocus sequence typing (MLST) was determined according to Tartof et al. [38]. Genes of* fosA*,* fosA3*,* fosC2* [22],* fosB2, fosC, fosX* [26],* fosB* [21],* fosA3/4 *[25], and* fosKP96* [24] were detected by PCR using the primers described previously. Gene of* fosA5* was detected by PCR using primer set: 5′-ACTGAATCACCTGACCCTGG-3′ and 5′-CGCATAATGGGTGTAGTCGC-3′. Full nucleotide sequences of six genes (*murA*,* uhpT*,* glpT*,* uhpA*,* ptsI*, and* cyaA*) were determined by a combination of direct sequencing and primer walking with the respective PCR products. PCR primer sequences are given in Table 1. The sequencing was performed with Big Dye Terminator Kit version 3.1 and 3730xI DNA analyzer (Applied Biosystems, Carlsbad, CA) at Hokkaido System Science (Sapporo, Japan).

### 2.5. Real-Time Reverse-Transcription (RT) PCR

*E. coli* cells were grown for 24 h in Luria-Bertani (LB) broth, and the cells were harvested and washed twice with M9 minimum salt solution. The suspended cells were used to inoculate to M9 minimum salt solution with or without 0.2% G6P supplementation and incubated for 30 min at 37°C. RNA was isolated from the cells using RNeasy Plus Mini Kit (Qiagen) according to the manufacturer's instructions. cDNA was prepared from the RNA using SuperScript III First Strand Synthesis Kit (Invitrogen, Carlsbad, CA) and random hexamer oligonucleotide primers. mRNA levels of* uhpT* and* rpoD *were quantified using QuantiFast SYBR Green PCR Mastermix (Qiagen) by LightCycler LC480 (Roche, Basel, Switzerland) with the cDNA as a template. PCR primers were given in Table 1. Levels of* uhpT *transcript were calculated by 2^−ΔΔct^ method, and data were normalized to the levels of the house-keeping gene* rpoD *mRNA.

## 3. Results

### 3.1. Antibiotic Susceptibility

**Figure 1:**
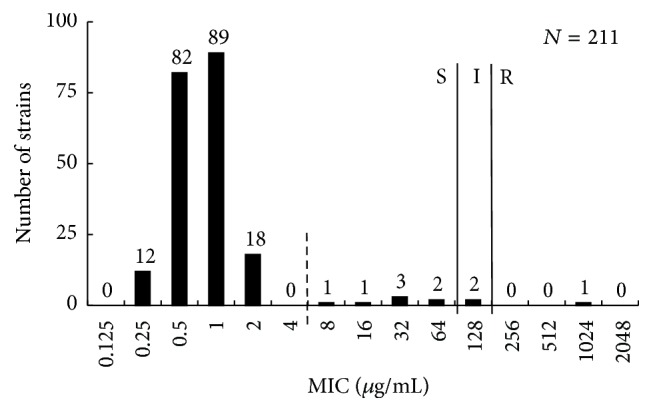
Distribution of fosfomycin MICs of the* E. coli* clinical isolates. The breakpoint is according to CLSI guideline. S: susceptible, I: intermediate, and R: resistant.

Antibiotic susceptibility was examined for 211* E. coli* clinical isolates. MICs were determined by broth microdilution method for LVX, GEN, IPM, and CAZ and by agar plate dilution method for FOM (Table 2). The distribution of FOM MIC was shown in Figure 1. Three strains (1.4%) were not susceptible, including resistant and intermediate. Furthermore, seven strains (3%) with elevated FOM MIC (≥8 *μ*g/mL, ≤64 *μ*g/mL) were observed out of normal distribution of the susceptible strains. FOM MICs of these ten strains were not affected by the presence or absence of G6P. By contrast, other susceptible strains (MIC ≤ 2 *μ*g/mL) showed an increase of FOM MICs in the absence of G6P (Table 3). Susceptibility to other antibiotics was examined for strains with reduced susceptibility to FOM (Table 2). LVX-resistant strains comprised 50% and GEN-resistant strains comprised 30% of these strains. IPM-resistant and CAZ-resistant strains were not observed; however 20% strains shared CTX-M-type ESBL genes. The occurrence of antimicrobial nonsusceptibility was not significantly high compared to total strains examined (data not shown). Genotypes (i.e., phylogenetic groups and MLST) and O-serogroups of these strains were variable (Table 3).

### 3.2. Analysis of Genes Associated with FOM Sensitivity

We examined molecular mechanisms underlying the reduction of FOM sensitivity (MIC ≥ 8 *μ*g/mL). First, genes encoding FOM-modifying enzymes were investigated. None of the following were detected in the strains examined:* fosA*,* fosA2 *to* fosA5*,* fosC*,* fosC2*,* fosB*,* fosB2*,* fosX*, and* fosKP96* (data not shown). Next, nucleotide sequences of* murA*,* uhpT*,* uhpA*,* glpT*,* ptsI*, and* cyaA* genes were determined (Table 3). No* murA* coding sequence mutations that would result in changes of amino acid residues in MurA were observed. A resistant strain (SRE257) and an intermediate-resistant strain (SRE91) had mutations in the genes that would result in deletions of a part of amino acid residues in UhpA and GlpT, respectively. In another intermediate-resistant strain (SRE49),* uhpA* and* uhpT* were not detected by PCR amplification with two distinct primer pairs (“full” and “partial” in Table 1). In one strain with MIC 32 *μ*g/mL (SRE252),* cyaA* was not detectable by PCR. All strains with reduced FOM MIC (≥8 *μ*g/mL) except one (SRE29), had several mutations in one or more genes leading to amino acid deletion or point mutation(s) of amino acid residues, compared with other susceptible strains.

### 3.3. Function and Expression of Carbohydrate Phosphate Transporters

To determine the activity of UhpT and GlpT, we examined cell growth in M9 minimum salt solution supplemented with G6P or G3P. Nine of ten strains with FOM MIC ≥ 8 *μ*g/mL did not grow within 24 h on G6P-containing M9 minimum salt agar. On the other hand, only three of ten strains did not grow in the presence of G3P (Table 3), and the two strains (SRE91 and SRE252) showed reduced MIC to FOM by addition of cAMP. This suggested that these two strains shared insufficient intracellular concentration of cAMP for full expression of GlpT and/or UhpT. These results suggested that FOM incorporation through the UhpT system is involved to a greater extent in the reduction of* E. coli *FOM compared to the GlpT system.

**Figure 2:**
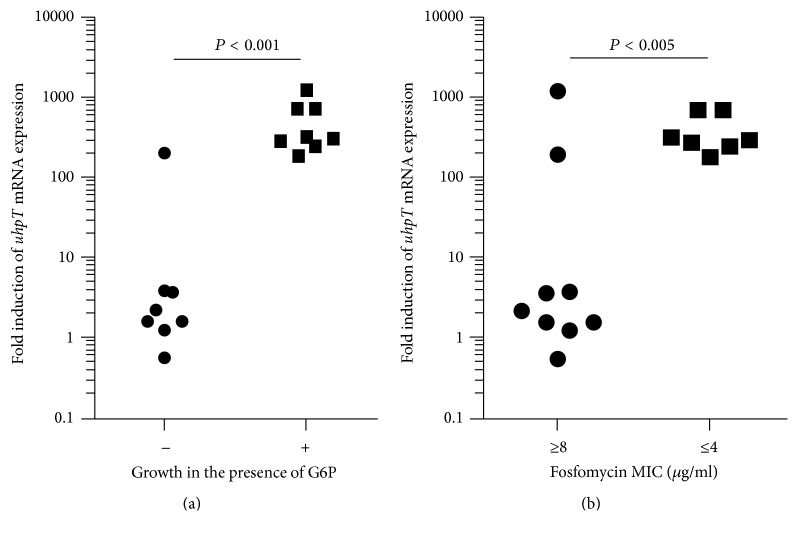
Induced expression levels of* uhpT* expression by the addition of G6P. Nine clinical isolates with reduced susceptibility to FOM; six susceptible clinical isolates and one standard strain are included. Data used to generate this figure are given in Table 2. Statistical significance was determined by Mann–Whitney test. (a) Comparison of strains not grown (−) or strains grown (+) in M9 minimum salt solution containing G6P. (b) Comparison of strains with FOM MIC ≥ 8 *μ*g/mL or ≤4 *μ*g/mL.

Expression of UhpT is induced by G6P [15–17]. We therefore determined* uhpT* gene induction by G6P using quantitative RT-PCR analysis (Table 3 and Figure 2). In strains with FOM MIC < 1 *μ*g/mL,* uhpT *expression was strongly induced (190- to 730-fold) by G6P. By contrast, in eight of ten strains with FOM MIC ≥ 8 *μ*g/mL, the* uhpT *induction was markedly lower (0.56- to 3.8-fold) or no* uhpT *signal was amplified by PCR. Of the remaining two strains, we observed a high induction (200-fold) of* uhpT* expression by G6P in SRE253 strain; however, the strain did not grow on G6P-containing M9 minimum agar. High induction (1200-fold) of* uhpT* by G6P was observed in SRE29 strain, and the strain grew on G6P-containing M9 minimum agar; however, it showed reduced susceptibility to FOM (MIC 32 *μ*g/mL). These results indicated that G6P-dependent growth lacked in most strains with reduced susceptibility to FOM because of lack of* uhpT* or a markedly reduced* uhpT* induction by G6P. However, the changes in* uhpT* expression and UhpT activity did not account for the reduced FOM susceptibility of a few strains (Figure 2).

## 4. Discussion

The frequency of FOM resistance has been recognized to be low. And it has been expected that the frequency of cross-resistance of FOM and other antibiotics is very low because of a unique mode of action. However, several reports examined FOM resistance in ESBL-producing* E. coli *[19, 23, 24, 28]. A report indicates that FOM-resistant and intermediate* E. coli* are more frequently resistant to other types of antimicrobials than FOM-susceptible strains [28]. These reports suggest the importance for surveillance of FOM-resistant* E. coli*, particularly for focus on their cross-resistance of FOM in multidrug resistance.

In this study, only one resistant and two intermediate strains were found among 211 clinical isolates from Japan (Figure 1). This indicated that FOM was a promising candidate agent against* E. coli* infections as generally described. However, we found seven strains susceptible according to the CLST breakpoint but reduced susceptibility (MIC ≥ 8 *μ*g/mL). Under the selective pressure of FOM usage, these strains might acquire FOM resistance more easily. In the present study, cross-resistance of strains with reduced FOM susceptibility to other antimicrobial drugs was not significantly higher than in other strains, and these strains were not concentrating on specific genotypes.

Based on previous reports, acquisition of FOM-modifying enzymes (encoded by* fos* genes) and mutations in* murA* gene results in resistance, that is, surpass of breakpoints, to FOM [18, 19, 28]. The present study did not identify such strains. Another resistance mechanism is altered FOM incorporation into bacterial cells. The G6P and G3P transporters contribute to incorporation of FOM into cells [13, 14]. Loss of function or decreased expression levels of them leads to a reduction of FOM susceptibility [18, 29, 39]. We found that loss of UhpT (G6P transporter) activity is more dominant than that of GlpT (G3P transporter) in strains with decreased FOM susceptibility. In resistant (>128 *μ*g/mL) and intermediate (128 *μ*g/mL) strains, we noted amino acid deletion in the respective encoded proteins and no PCR amplification of the transporter-related genes (Table 3). In strains with FOM MICs between 8 and 64 *μ*g/mL, gene mutation(s) leading to alternations of amino acid residues were found; however, it was unclear whether these contributed to the reduced susceptibility. Among the ten strains with reduced susceptibility, nine strains did not grow on G6P-supplemented M9 minimum salt agar. This suggested that in these nine strains G6P-induced UhpT function was attenuated. We found several mutations in* cyaA* and* ptsI* in some of these strains. Dysfunction of CyaA and PtsI leads to decrease in intracellular concentration of cAMP and insufficient expression of GlpT and UhpT [16, 17, 31]. Only two strains (SRE91 and SRE252) with reduced susceptibility to FOM showed that MIC was decreased by the exogenous addition of cAMP, and these strains did not grow in M9 medium supplement with G3P. Certainly, SRE252 defected* cyaA*; however, SRE91 did not share any mutations in* cyaA* and* pstI*. Thus reduced susceptibility to FOM in the other strains was not explained by the insufficient intracellular concentration of cAMP caused by dysfunction of* cyaA* and* pstI*. The mutations in these genes found in this study (Table 3) seemed to scarcely relate with dysfunction of cAMP synthesis.

In conclusion, FOM resistance occurs with low frequency and is independent of resistance to other antimicrobials in* E. coli* clinical isolates from Japan. On the other hand, we identified strains with decreased FOM susceptibility. Most of them displayed fluctuated activity of the G6P transporter UhpT. However, G6P transporter function was altered even though the G6P-induced uhpT expression and amino acid sequence of UhpT were preserved in one strain (FOM MIC 8 *μ*g/mL). Another strain (FOM MIC 32 *μ*g/mL) displayed reduced susceptibility to FOM and no alteration of MIC in the presence and absence of G6P, even though G6P-induced* uhpT* expression, amino acid sequence of UhpT, and growth on G6P-supplemented M9 minimum salt agar were preserved. The exact molecular mechanism of the reduced susceptibility of these strains remains unclear and requires further evaluation.

## Figures and Tables

**Table 1:** PCR and real-time RT-PCR primers used in this study.

Primer		Sequence (5′-3′)	Reference
*murA-full* ^a^	F	AAACAGCAGACGGTCTATGG	[18]
	R	CCATGAGTTTATCGACAGAACG	
*glpT-full*	F	GCGAGTCGCGAGTTTTCATTG	[18]
	R	GGCAAATATCCACTGGCACC	
*uhpT-full*	F	TTTTTGAACGCCCAGACACC	[18]
	R	AGTCAGGGGCTATTTGATGG	
*uhpT-partial*	F	ATGCTGGCTTTCTTAAACC	[19]
	R	TTATGCCACTGTCAACTGC	
*uhpA-full*	F	GATCGCGGTGTTTTTTCAG	[18]
	R	GATACTCCACAGGCAAAACC	
*uhpA-partial*	F	ATCACCGTTGCCCTTATAGA	[19]
	R	TCACCAGCCATCAAACAT	
*ptsI-full*	F	GAAAGCGGTTGAACATCTGG	[18]
	R	TCCTTCTTGTCGTCGGAAAC	
*cyaA-full*	F	AACCAGGCGCGAAAAGTGG	[18]
	R	ACCTTCTGGGATTTGCTGG	
*rpoD-qPCR*	F	CAAGCCGTGGTCGGAAAA	[20]
	R	GGGCGCGATGCACTTCT	
*uhpT-qPCR*	F	AAGCCGACCCTGGACCTT	[20]
	R	ACGGTTTGAACCACATTTTGC	

^a^Primers designated “full” were used for direct sequencing, and “qPCR” were used for real-time RT-PCR.

**Table 2:** Antibiotic susceptibility and the presence of ESBL genes in *E.coli* strains with FOM MIC ≥ 8 *μ*g/mL.

Strains	MIC (*μ*g/mL)					ESBL^a^gene
	Fosfomycin (≥128)^b^	Levofloxacin (≥4)^b^	Gentamicin (≥8)^b^	Imipenem (≥2)^b^	Ceftazidime (≥8)^b^	
SRE257	1024^*∗*^	≤0.125	1	0.125	0.125	—
SRE91	128^#^	32^*∗*^	4	0.25	0.125	—
SRE49	128^#^	16^*∗*^	2	0.125	0.25	—
SRE54	64	≥64^*∗*^	≥64^*∗*^	0.125	2	CTX-M14
SRE237	64	≤0.125	4	0.125	0.125	—
SRE29	32	16^*∗*^	≥64^*∗*^	0.125	0.125	—
SRE252	32	0.5	2	0.125	0.125	—
SRE280	32	≤0.125	8^*∗*^	0.5	0.125	—
SRE18	16	32^*∗*^	2	0.125	2	CTX-M2
SRE253	8	≤0.125	4	0.5	0.125	—

^a^ESBL: extended spectrum *β*-lactamase.

^b^Breakpoints (*μ*g/mL) are according to CLSI.

^*∗*^Resistant.

^#^Intermediate.

**Table 3:** Characteristics of *E. coli* strains used in the study.

Strain	Specimen	Sero- group	Phylo- genetic group	MLST	MIC^a^(*μ*g/mL)			Growth on M9 agar supplemented with:		*uhpT* expression induced by G6P^b^	Amino acid residue alternations in proteins encoded by *glpT*, *ptsI*, *cyaA*, *murA*, *uhpA*, and *uhpT* genes^c^					
					G6P −	G6P +	G6P+ cAMP+	G3P	G6P		*cyaA*	*glpT*	*murA*	*ptsI*	*uhpA*	*uhpT*
SRE257	Urine	O1	B2	95	1024	1024	1024	+	−	1.24	—	—	—	Val399Leu	163~188 deletion	—
SRE91	Aspiration tube	O1	D	648	128	128	32	−	−	3.78	—	155~158 deletion Phe176Leu	—	—	Thr3Ala	—
SRE49	Urine	O25b:H4	B2	131	128	128	128	+	−	NT	His716Leu	—	—	Lys410Arg	ND	ND
SRE54	Urine	O25b:H4	B2	131	64	64	64	+	−	1.59	His716Leu	—	—	Ala443Thr Gly452Asp	—	—
SRE237	Urine	O25b:H4	B2	131	64	64	64	+	−	1.59	His716Leu	—	—	—	—	—
SRE29	Urine	O25b:H4	B2	131	32	32	32	+	+	1226.22	—	—	—	—	—	—
SRE252	Urine	O25a	D	73	32	32	4	−	−	3.68	ND	Ile171Thr	—	Lys145Asn	—	—
SRE280	Ascites	O12	D	1486	32	32	32	+	−	0.56	Ser142Asn	—	—	—	—	—
SRE18	Urine	ND	D	405	16	16	16	+	−	2.22	—	—	—	—	Met1Ile	—
SRE253	Urine	O18	B2	95	8	8	8	−	−	198.09	His716Leu	—	—	—	—	—
SRE40	Decubitus	O25a	D	501	32	0.5	NT	+	+	284.05						
SRE41	Catheter urine	O1	D	648	8	0.5	NT	+	+	328.56						
SRE110	Catheter urine	O25b:H4	B2	131	8	0.5	NT	+	+	190.02						
SRE205	Urine	ND	A	131	8	0.5	NT	+	+	734.19						
SRE227	Pus	O1	B2	95	8	0.5	NT	+	+	247.28						
SRE30	Urine	O1	D	648	8	0.25	NT	+	+	719.08						
ATCC 25922		NT	NT		32	0.5	NT	+	+	308.69						

^a^FOM MICs were determined in the presence (+) or absence (−) of glucose-6-phosphate (G6P) and/or cAMP.

^b^ *E. coli *cells were incubated in M9 minimum salt solution in the presence or absence of G6P. The *uhpT* mRNA levels were determined by real-time RT-PCR, and the data were normalized to *rpoD* mRNA levels. Induction of *uhpT* expression by G6P was calculated by dividing the *uhpT *mRNA level in the presence of G6P by the *uhpT* mRNA level in the absence of G6P.

^c^Amino acid mutations found only in strains with reduced FOM susceptibility (MIC ≥ 8 *μ*g/mL) compared with strains with FOM MIC < 1 *μ*g/mL.

ND: not detected. NT: not tested.
